# Anti-Exceptionalism About Requirements of Epistemic Rationality

**DOI:** 10.1007/s12136-020-00450-0

**Published:** 2020-09-26

**Authors:** Claire Field

**Affiliations:** Department of Philosophy, University of Stirling, Stirling, UK

**Keywords:** Rational requirements, Fixed point thesis, A priori, Misleading evidence

## Abstract

I argue for the unexceptionality of evidence about what rationality requires. Specifically, I argue that, as for other topics, one’s total evidence can sometimes support false beliefs about this. Despite being prima facie innocuous, a number of philosophers have recently denied this. Some have argued that the facts about what rationality requires are highly dependent on the agent’s situation and change depending on what that situation is like (Bradley, *Philosophers’ Imprint*, *19*(3). 2019). Others have argued that a particular subset of normative truths, those concerning what epistemic rationality requires, have the special property of being ‘fixed points’—it is impossible to have total evidence that supports false belief about them (Smithies, *Philosophy and Phenomenological Research*, 85(2). 2012; Titelbaum 2015). Each of these kinds of exceptionality permits a solution to downstream theoretical problems that arise from the possibility of evidence supporting false belief about requirements of rationality. However, as I argue here, they incur heavy explanatory burdens that we should avoid.

A natural thought is that evidence about what rationality requires behaves in roughly the same way as evidence about other topics. If so, then your evidence can sometimes support false beliefs about what rationality requires. A number of philosophers have recently denied this. Some because they have thought that the facts about what rationality requires change depending on the agent’s evidential situation (Bradley 2019; Kvanvig 2014; Way and Whiting 2016). Others because they have thought that normative truths concerning what epistemic rationality requires have the special property of being ‘fixed points’: every evidential situation justifies true belief about them (Smithies 2012; Titelbaum 2015). These are two different ways to defend the Impossibility Thesis.

- *Impossibility Thesis*: Evidential situations requiring false belief about what rationality requires are impossible.

One reason to like the Impossibility Thesis is that it rules out an awkward evidential situation that threatens the consistency of our theories of epistemic rationality. Suppose that rationality requires you to believe P. Then, suppose that you have some evidence supporting the false belief that, in fact, you are required to *not* believe P. Are you required to believe P, or not? On the one hand, you seem to be required to *not* believe P, because this is what your evidence about what rationality requires supports. On the other hand, you seem to be required to believe P, since this is what the true requirements in fact require. You are in a bind—you appear to be required to both believe and *not* believe P. The Impossibility Thesis offers a way out—it rules out situations in which this inconsistency arises. However, it creates more problems than it solves. I argue that we should prefer the following Simple View of evidential support.

- *Simple View*: Evidential situations supporting false belief are possible about all topics.

Following others in this debate, I understand requirements of rationality as necessary conditions of having rational epistemic attitudes, without taking much of a stand on what these are.^1^ However, I do assume that there are some necessary conditions of rationality and that there is a fact of the matter about what these are.

Section 1 outlines possible situations in which an evidential situation appears to support false belief about what rationality requires and shows how the Simple View can explain it easily. Section 2 examines Perspectival defences of the Impossibility Thesis, according to which evidence about what rationality requires changes the facts about what rationality requires. Section 3 examines Objectivist defences of the Impossibility Thesis, on which evidential situations can never support false beliefs about what rationality requires. Section 4 outlines some reasons for optimism about dealing with the problem of inconsistent requirements without the Impossibility Thesis.

## Misleading Evidence

Evidence can support false belief about all kinds of subject matters. The Simple View says that what rationality requires is a subject matter like any other, and so something that evidence can support false belief about. Studying philosophy is a good way to get evidence for false belief about what rationality requires. Consider:

- *Logic 101*. Suppose that it is a requirement of rationality to avoid contradictory belief. Suppose also that you are taking your first introductory course in logic. Unfortunately, your instructor is an overzealous advocate of dialetheism and believes that rationality sometimes requires contradictory belief, for example in response to instances of the Liar Paradox.^2^ He well-meaningly introduces you to all the best arguments for dialetheism, some paraconsistent logic, and some unconvincing challenges to dialetheism. By the end of the course, you believe, falsely, that rationality sometimes requires contradictory belief.

A natural explanation of this situation is that your evidence supports a false belief about what rationality requires. Your instructor counts as an expert in this domain, which makes his testimony evidence. The philosophical arguments you study also give you evidence. You lack the capacity to refute any of this evidence. However, none of this changes the facts about what rationality requires—rationality still requires that you avoid contradictory belief.

Similar cases are possible for any other putative requirement of rationality. Epistemologists are in the business of making arguments for and against various requirements of rationality: requirements to believe/not believe lottery propositions,^3^ requirements to conciliate^4^/remain steadfast^5^ in the face of peer disagreement, requirements to be level coherent,^6^ or not.^7^ The extent of apparently reasonable disagreement about what rationality requires suggests that false belief about what rationality requires is common and that it is often evidentially supported.

This simple explanation is ruled out by the Impossibility Thesis. As I argue in the following sections, neither of the ways of defending the Impossibility Thesis offers an adequate alternative explanation of situations like Logic 101.

## Perspectivism

Perspectivism holds that what rationality requires depends on the agent’s evidential situation. For example, Bradley (2019) avoids the problem of inconsistent requirements by arguing that evidence against a putative requirement changes what is required.^8^ If your situation includes evidence that a rule does not apply, then that rule does not apply—the rule is defeated. On this view, the Impossibility Thesis is true because if your evidential situation indicates that rationality requires P, then rationality really does require P.

Bradley focuses on epistemic rules such as ‘believe P if you have a perception that P’ and ‘believe P if you have testimony that P’. He argues that evidence that one of these rules does not apply in a particular case defeats that rule—it no longer applies in that case. Instead, what each agent is required to believe in a situation is specified by a complicated and indefeasible ‘Uber-rule’. However, an important shared feature of the rules that Bradley discusses is that they gain respectability from the more fundamental epistemic rule ‘believe what the evidence supports’. If this is right, then it is not surprising that evidence against the reliability of one’s perceptions can defeat the rule. The rule only ever had instrumental force—it was a good way of complying with the fundamental rule to believe what the evidence supports. Receiving evidence that trusting perception is not a way to believe what the evidence supports *does* defeat the perceptual epistemic rule, but it is less clear that other, non-evidential, fundamental rules can be defeated so easily. Rational requirements are more fundamental than instrumental rules like ‘believe P if you have a perception that P’, and they need not be evidential.^9^

There are two options for interpreting how Bradley should respond to this point.^10^ Either he must concede that there are some fixed points that one cannot have misleading evidence about, namely the more fundamental requirements of rationality, whatever those are.^11^ This would convert the view from a Perspectivist to an Objectivist view. Or, he could say that the more fundamental requirements of rationality are also defeasible—that is, affected by the agent’s evidence. On this interpretation, the only true requirement is a long and complicated ‘Uber-rule’ specifying very precisely what is required of each agent in each situation.

If this is the position, then it is not as good an explanation as the Simple View of cases such as Logic 101. There are three key reasons for this. First, it amounts to an error theory about the more familiar rational requirements—requirements such as the Law of Non-Contradiction were never universally applicable requirements applying to all rational agents, as we might have thought they were. Logic 101 is not a case in which you are misled by evidence for a false view, but rather a case in which, in fact, you are required to believe a contradiction. On this view, requirements of rationality are flimsier than we might have thought. They are also not as good guides as we might have hoped. Bradley accepts this (2019: 14), but it is nevertheless a cost that must be justified by robust theoretical gains.^12^

Second, this view faces pressure to restrict what can count as rationally required in such a way that avoids collapsing into triviality, where any attitude at all can potentially be rationally required. It is unclear whether this can be done while maintaining commitment to the view that your evidential situation determines what rationality requires. This is particularly true if one shares Bradley’s view that something’s seeming to be evidence is sufficient for it to be evidence—for example, he expresses incredulity at the view that “what seems like evidence doesn’t even count as evidence” (2019: 6). However, what our evidence is and what our evidence seems to be are very different matters. Something may appear to be evidence from your perspective, even if it is not^13^^,^^14^. However, introducing restrictions on which attitudes could be rationally required risks compromising the view’s core commitments and ruling out possible evidential situations. This is an Objectivist rather than a Perspectivist strategy.

Third, these awkward theoretical features are introduced unnecessarily. Bradley is right to point out that there is something positively evaluable about agents who have evidence that a rule does not apply and do not follow that rule. However, it does not follow from this that the rule ceases to apply to the agent in that case. Agents can break rules and be excused or even justified. Excuse and justification are positive evaluations in the sense that they remove blame that would have been deserved had the rule been broken without excuse or justification.^15^ However, they do not imply that the rule ceases to apply. So, we can explain the cases Bradley discusses without postulating requirements of rationality that fluctuate in response to details of the agent’s situation. This offers another way to describe Logic 101: their misleading evidence gives them an excuse for disobeying the requirements of rationality, but it does not make it rational for them to disobey the requirements.^16^ The following section examines Objectivist defences of the Impossibility Thesis.

## Objectivism

Objectivism defends the Impossibility Thesis by making a claim about which evidential situations are possible. Specifically, it rules out the possibility of situations in which the total evidence supports false belief about what rationality requires.

One way that some philosophers have attempted to rule out these situations is by appeal to the agent’s already having strong evidence supporting the truth about what rationality requires. For example, Smithies claims that “one always has a special kind of epistemic access to facts about which propositions one has justification to believe” (2012: 273), and Titelbaum that "[E]very agent possesses a priori, propositional justification for true beliefs about the requirements of rationality in her current situation" (2015: 276). Call this claim Assets.

- *Assets:* All agents, in all possible situations, have evidence that overwhelmingly supports the truth about what rationality requires.

Smithies and Titelbaum take Assets to support the Impossibility Thesis, because they take it to rule out situations in which the agent has total evidence that supports a false belief about what rationality requires.

One possible motivation for thinking that Assets is true is that it is a consequence of Bayesian commitments to logical omniscience. Logical omniscience says that for all agents in all situations, their evidence—whatever it is—supports all logical truths to credence 1. Some have thought that our a priori evidence is identical to that of the Bayesian ideally rational agent (Christensen (2004: 162), Smithies (2012: 8)). However, logical omniscience is often, if not usually, taken to be a bug rather than a feature of Bayesian epistemology.^17^ Commitment to logical omniscience introduces the significant challenge of working out how to make intelligible the claim that non-ideal agents have this evidence supporting credence 1 in all logical truths, even when this is opaque to them.^18^ Not only this, but we would need a good story about how logical truths are related to requirements of rationality.^19^

Some have thought that Assets is implied by the unrestrictedness of a priori reasoning. As Titelbaum goes on to explain, “[a]n agent can reflect on her situation and come to recognize facts about what that situation rationally requires. Not only does this reflection provide her with justification to believe those facts; that justification is ultimately empirically indefeasible.” (2015: 276). One might think that because the question of what rationality requires is a priori, so long as agents have the ability to reflect, then they can easily acquire evidence for the truth about what rationality requires. While there are physical limitations on, for example, where I can transport my body to gather empirical evidence, there are no such limitations on what I can think about.^20^ However, it is not clear that the sense in which agents in some situations ‘could’ come to acquire evidence for normative truths about rationality is sufficient to establish Assets. For example, consider agents who have never considered the matter, who have apparent defeaters or rebutters that count against the normative truths, or who lack the intellectual competence to do the necessary reasoning. If there is a sense in which these agents *could* acquire evidence for the truth, is this a sense of ‘could’ that affects what they are now justified in believing? This is not clear.

Even if true, Assets cannot establish the Impossibility Thesis alone. Assets says that we always have evidence that overwhelmingly supports the truth about what rationality requires. To get from this to the Impossibility Thesis, it must also be true that: this evidence is not defeasible by other features of the agent’s situation, and this evidence plays a meaningful role in determining what the agent ought to believe. In other words, Assets needs help from the following two claims:

- *Indefeasibility*^21^: All agents, in all situations, have evidence for the truth about what rationality requires that is indefeasible.
- *Sufficiency*: All agents, in all situations, have evidence for the truth about what rationality requires that is sufficient to rule out the possibility that their evidential situation requires false beliefs about what rationality requires.

If all three of Assets, Indefeasibility, and Sufficiency were true, then the Impossibility Thesis would be true, because our total evidence would always require believing the truth about what rationality requires. All three claims are contentious. We have already seen some reasons to worry about Assets. As the following subsections outline, Indefeasibility and Sufficiency are also dubious. Even worse, this strategy rules out the simple explanation of cases of apparent misleading evidence about what rationality requires.

### Indefeasibility

To get from the Assets claim to the Impossibility Thesis, our putative evidence for the truth about what rationality requires would need to be indefeasible. However, cases such as Logic 101 look like cases in which if there ever were any Assets, they are defeated. So, if we do have evidence that overwhelmingly supports true beliefs about what rationality requires, it is not clear that this evidence is *indefeasible.*^22^

For example, suppose, as some have thought, that we have default justification for basic laws of logic such as the Law of Non-Contradiction.^23^ In some sense, they are such that they are impossible to rationally doubt. If this is right, then one might be tempted to think that in Logic 101, your evidence could not really support believing that rationality sometimes requires contradictory belief—you must be confused. However, you are a beginner. Although you may start out with default justification for the truth about what rationality requires, it is much less clear that this justification remains undefeated in the face of all the testimonial and argumentative evidence you receive against it. It is not at all clear that your default justification can ensure that your evidential situation decisively supports believing the truth about what rationality requires. It may do this in ordinary circumstances, but in ordinary circumstances, you are not given convincing arguments against it. As a beginner, you have nothing to back it up with. When you begin to acquire more and more evidence in favour of the false view about what rationality requires, your evidence begins to tilt in that direction.

According to standard accounts of defeat, justification can be defeated by both undermining and rebutting evidence, regardless of the strength of the justification (Brown 2018; Pollock 1986). So, not only are you justified in believing the false view about what rationality requires, but this justification also defeats any other justification you could have been presumed to have for the truth about what rationality requires.^24^ If this standard view is right, then it cannot be true that our justificatory Assets are indefeasible.

Indefeasibility does not simply fall out of the apriority of the Assets, as some have thought. A priori justification is, by definition, justified independent of experience. Some have taken this to imply that any misleading evidence one could acquire for a priori claims must be, in some sense, experiential. For example, Smithies makes the claim that “a priori justification for beliefs about logic has its source in logical facts, rather than psychological facts about experience, reasoning, or understanding.” (2015: 2270). Since misleading evidence about logic cannot be a ‘logical fact’, its source must, on this view, be a ‘psychological fact about experience, reasoning, or understanding’.^25^ From this, we can build the following argument for the Indefeasibility claim:

1. If P is justified a priori, then it is justified independent of any experience.
2. If justification is independent of experience, then it cannot be defeated by experience.
3. The misleading evidence in the cases is derived from experience.
4. So, misleading evidence cannot defeat our propositional justification for the truth about what rationality requires (indefeasibility).

However, both premises 2 and 3 are contentious. While you may have been justified in believing that rationality prohibits contradictions before you took Logic 101,^26^ it is implausible that you are still justified in believing this after you have had heard the professor’s testimony. The fact that hearing the testimony is an experience is irrelevant to whether it defeats your justification.^27^

For premise 3 to be true, we need to assume a contentious understanding of ‘experience’, on which thinking through philosophical arguments is a (non-a priori) experience. On this contentious view, propositions rather than processes are a priori. Episodes of thinking that stray from logical and philosophical truth are not a priori, but rather experiential.^28^ There are two problems with assuming this contentious view of experience. Firstly, drawing a line between the a priori and the a posteriori is notoriously difficult. A prima facie plausible alternative view says that processes can be a priori, so that any evidence one acquires through a priori reasoning is itself a priori, and beliefs supported by that a priori evidence can be justified a priori.^29^ So, defending Indefeasibility by citing that the justification is a priori would mean assuming one way of drawing the a priori/a posteriori distinction - being a priori implies being true.

Not only this, but this way of drawing the distinction means taking a disjunctive view of abstract thinking—particular instances of abstract thinking will have very different epistemic statuses depending on their truth value. When abstract reasoning results in truth, one is a priori justified. When it goes wrong, the reasoning was not a priori at all, merely empirical or experiential. Sturgeon argues against disjunctivism about visual experience (1998: 182). He points out that veridical, illusory, and hallucinatory experiences have various features in common, and we should want a theory that can explain these common features. However, disjunctivism precludes an explanation of these common features—it maintains that they are distinct in nature. I am inclined to agree with Sturgeon that a view that can explain common features is preferable, ceteris paribus, to a view that cannot.

Moreover, these points may turn out to be irrelevant. Theories of what rationality requires may not, in fact, be justified a priori. Anti-exceptionalist accounts of logic deny that logical theories are not justified a priori.^30^ Instead, they are justified abductively. If this is right, then agents do not need a priori justification to be justified in their beliefs about logic. If this is true of logic, then it may well also be true for theories of what rationality requires.^31^

More importantly, Indefeasibility leaves us without a good explanation for cases in which our putative Assets appear to be defeated. Some have attempted to explain cases of apparent defeat using a distinction between defeat and some weaker defeat-like status. They have argued that although our Assets cannot be defeated, they can be ‘disabled’ (Smithies 2015), or the agent can be ‘rationally compromised’ (Ichikawa and Jarvis 2013). Such views say that when S’s justification for P is disabled, S is unable to use that justification in reasoning and unable to justify belief that P on the basis of that justification. Nevertheless, S’s justification for P is not defeated. Unfortunately, this strategy permits only implausible explanations of the target cases.^32^

Views that make use of disabling typically recognise only two ways that a priori propositional justification can be disabled. Either the agent makes an error due to cognitive incapacity akin to Chomskian performance limitations or the agent has empirical evidence that she currently lacks the cognitive capacity to reason successfully. Limitation in cognitive processing capacity is an example of genuine cognitive incapacity. Some logical truths are too complicated to be deduced by ordinary human agents, even though they are entailed by their evidence, and propositions they already believe.^33^ Cognitive biases are another example. They can cause performance error in reasoning by distracting agents from the facts they know to be true or would assert on reflection. Typical errors such as the conjunction fallacy are often like this. Distracted by irrelevant information, many people who should know better will claim that a conjunction is more likely than one of its conjuncts alone.^34^ Disabling allows the agent to retain her propositional justification, even while it is disabled by cognitive performance limitations that prevent her at that moment from drawing the correct conclusion.

However, this cannot explain cases like Logic 101. Here, your evidential situation supports the false belief about rationality because you are exercising your cognitive faculties *well*. Had you exercised them less well, you may not have followed the arguments, and so likely would have avoided acquiring evidence for the false belief about rationality. A possible line of response to this is that in Logic 101, your faculties are limited in one sense—your capacities for abstract philosophical reasoning are those of a beginner. Not only this, but you are being taught incorrect rules about how to reason rationally. There are, perhaps, many other good things to say about your reasoning capacities, but they are limited in some important respects. However, this response does not easily transfer to cases involving non-logical requirements of rationality, such as requirements to conciliate (or not) in response to peer disagreement. When you have false beliefs are about these, it is much less plausible that the mere fact that you endorse a false view compromises your general capacities for reasoning. If it did, this point would overgeneralise. It would also apply to experienced philosophers who hold false views about what rationality requires, but their reasoning capacities are clearly not limited.^35^

One might reply that ideal agents would nevertheless have no false beliefs about what rationality requires. However, using the ideal to define what counts as a cognitive limitation risks generating a theory that is irrefutable. If every false belief about what rationality requires were an instance of limited capacity in which the agent *really* had (disabled) propositional justification for the truth, then the claim that an agent has propositional justification for P would be irrefutable, and ultimately trivial.

Nor can *evidence of* cognitive incapacity explain the cases. This is the remaining option on offer for how Assets could be disabled, as in the following example:

- *Coffee*. Suppose that I work out my proof of T after having coffee with my friend Jocko. Palms sweaty with the excitement of logical progress, I check my work several times, and decide that the proof is good. But then a trusted colleague walks in and tells me that Jocko has been surreptitiously slipping a reason-distorting drug into people’s coffee—a drug whose effects include a strong propensity to reasoning errors in 99% of those who have been dosed (1% of the population happen to be immune). He tells me that those who have been impaired do not notice any difficulties with their own cognition—they just make mistakes; indeed, the only change most of them notice is unusually sweaty palms. (Christensen 2007: 3).

According to disabling views, evidence that my cognitive abilities are impaired prevents me making the logical inferences licenced by my propositional justification. It does this without affecting my propositional justification. Were this justification not disabled, I could use it to draw the correct inferences.

There are important differences between Coffee and Logic 101. In Coffee, I receive empirical evidence about my logical reasoning capacities are impaired. Responding appropriately to this means giving appropriate (i.e. significantly less, or no) weight to evidence gathered using those capacities. Dealing properly with the evidence gathered from my capacities prevents me from forming justified beliefs based on my propositional justification for the logical facts. This is true for any other piece of logical reasoning I might undertake while I have evidence that I am under the influence of the drug. In contrast, in Logic 101, your evidence directly concerns what rationality requires. Responding appropriately to it means weighing it in your deliberation about the truth of what rationality requires, against other evidence that you have about this topic. This is quite different. This suggests that Logic 101 is not a case of disabling via evidence of cognitive capacity.

So, Logic 101 does not seem to be a case of disabling. However, we might think there is another explanation of it available to Objectivists, namely that your evidence does not support the false belief about what rationality requires because the very process of weighing up what your evidence supports relies upon classical reasoning principles, and by extension the Law of Non-Contradiction. One might worry that this dependence undermines the evidence that you seem to have for the false belief that rationality sometimes requires contradictory belief.^36^ If this evidence really depends implicitly on classical logic,^37^ including the Law of Non-Contradiction, one might think that this just provides more evidence for the Law of Non-Contradiction and against the false view that rationality sometimes requires contradictory belief.

It is worth pointing out that this explanation, even if correct, would only rule out cases of misleading evidence about requirements of rationality that are sufficiently close to principles of logic. It will not apply to evidence about requirements to conciliate in response to peer disagreement, or avoid believing lottery propositions. There is also some distance between the claim that one’s evidence supports or relies upon some logical principle, and the claim that it supports or relies on a requirement of rationality based on that logical principle. We cannot assume a straightforward relationship between the truths of logic and truths about what rationality requires. For example, while (P & (P → Q)) → Q) is a logical truth, it is not a demand of rationality that if one believes P, and believes that (if P, then Q), then one also believes Q. Furthermore, even if there were a straightforward relationship between these, it is not clear that this would tip the evidential scales in favour of believing the true rational requirements, because it is not clear that the logical or rational principles we depend upon to acquire evidence *themselves *count as part of our evidence. This is uncontroversial in other domains – compare our beliefs about the physics of middle sized objects. I believe that if I drop my coffee mug, it will fall to the ground. My evidence for this consists in past observations of dropping mugs, and some elementary and incomplete understanding of gravitational laws. However, its truth is underpinned by various more complicated principles of physics that I have never considered and would not understand if I did. These more complicated principles of physics do not form part of my evidence, but we might think that my reasoning about the physics of middle-sized objects implicates them in an analogous way that your reasoning in Logic 101 implicates principles of classical logic. However, my evidence about whether mugs will fall does not support the complicated principles of physics, and I do not have evidence that supports believing these principles—at least not unless I learn much more about physics. So, even if it is true that ordinary reasoning about evidence is underpinned by principles of classical logic, these principles are not part of your evidence in Logic 101, at least in the absence of further arguments, evidence, and understanding of logic. This is true even assuming that you exhibit tendencies to avoid contradictory belief. Once you have fully absorbed the arguments you study for dialetheism, you have evidence that this tendency is *only* a tendency and not supported by any requirement of rationality.

So, Indefeasibility does not seem to be true. Moreover, the explanations of cases like Logic 101 that are compatible with Assets are inadequate. We should thus prefer the Simple View - evidential situations supporting false belief are possible about all topics, including what rationality requires. However, even if we did not prefer the Simple View, there is a further problem with Assets—it needs Sufficiency, and Sufficiency is false.

### Sufficiency

In addition to Indefeasibility, defending the Impossibility Thesis via Assets requires Sufficiency.

- *Sufficiency:* All agents, in all situations, have evidence for the truth about what rationality requires that is sufficient to rule out the possibility that their evidential situation requires false beliefs about what rationality requires.

Sufficiency is importantly distinct from Indefeasibility. Indefeasibility is a claim about the *strength* of evidence. It says that the evidence postulated by Assets is maximally strong, such that it cannot be defeated. Sufficiency is a claim about how evidence, whatever its strength, can be used in deliberation. It says that the evidence postulated by Assets *rules out* other beliefs. The claims may appear similar because when indefeasible evidence for P does bear on what S ought to believe, it will typically rule out beliefs that not-P. However, Indefeasibility does not by itself imply the ability to rule out beliefs to the contrary. That the evidence bears on what S ought to believe is a claim strictly independent of Indefeasibility. Without Sufficiency, even if it were true that all agents have evidence supporting the truth about what rationality requires, and that this evidence was indefeasible, further argument would be required to establish how and why our having this evidence rules out the possibility of one’s total evidence supporting false beliefs about rationality, as the Impossibility Thesis claims.

For evidence to be able to rule out the possibility of one’s situation requiring false belief about what rationality requires, it would need to play a role in determining what the agent ought to believe. However, it does not seem that it does.

The problem is that the justification that Assets provide is propositional, but not necessarily doxastic. Assets say that justification for the requirements of rationality is available to all rational agents in all situations, but this implies only that were they to reflect properly, then they would be rational to believe the truth about what rationality requires. This is a point on which Objectivist theories that postulate Assets agree. Here is Titelbaum again:

How is the justificatory map arranged such that one is never all-things-considered justified in both an attitude A and the belief that A is rationally forbidden in one’s current situation? The most obvious answer is that every agent possesses a priori, propositional justification for true beliefs about the requirements of rationality in her current situation.^48^ An agent can reflect on her situation and come to recognize facts about what that situation rationally requires. Not only can this reflection justify her in believing those facts; the resulting justification is also empirically indefeasible (2015: 276).

Smithies qualifies Assets as follows:

[Assets] is not a doxastic thesis about which of one’s beliefs are justified, but is rather an epistemic thesis about which propositions one has justification to believe. Justified belief requires not only having justification to believe a proposition, but also using it in believing that proposition on the basis of one’s justification to believe it. By contrast, having justification to believe a proposition does not require using it in forming a justified belief. As we shall see, this distinction between epistemic and doxastic versions of the accessibility thesis is crucial for avoiding the problems of over-intellectualization and vicious regress (2012: 276).

Restricting Assets to a claim about propositional justification can seem to remove some of the pressure to accommodate or explain cases like Logic 101. The idea is that in such cases, the agent is propositionally justified in the truth in spite of their misleading evidential situation. This can then be used to deny the claim that agents in such cases would be rational to believe falsely about rationality, and so avoid the problem of inconsistent requirements. However, this claim is silent on what agents are doxastically justified in believing, and this silence threatens the legitimacy of the restriction.

There are three options for what you are doxastically justified in believing in Logic 101. Perhaps the most natural option is that you are doxastically justified in believing falsely about what rationality requires. This, after all, is what your evidence appears to support. However, if this is right, then it is less plausible that you are propositionally justified in believing the truth about what rationality requires. Traditionally, P is propositionally justified for S if and only if S has justifiers J sufficient to justify P.^38^ Standardly, S is doxastically justified in believing P when the following three conditions hold:

- P is propositionally justified for S.
- S believes P.
- S believes P on the basis of her propositional justification.

On this view, doxastic justification implies propositional justification. So, if you are doxastically justified in believing some false claim about what rationality requires, P, and you are propositionally justified in believing the true claim about rationality, not-P, then it is not true that the evidence postulated in Assets rules out evidential situations that require false beliefs about what rationality requires.

This seems to be the situation in Logic 101. The proposition that rationality sometimes requires contradictory belief is supported by justifiers that you have—the evidence you acquire by taking Logic 101, albeit not to a maximal degree. So, in Logic 101, you meet all of (a)–(c). You believe it, you believe it on the basis of justifiers (i.e. evidence), and your justifiers support the truth of the claim. So, given the ‘orthodox view’ of the relationship between doxastic and propositional justification, you are both doxastically and propositionally justified in the false belief about what rationality requires. On this first option, Sufficiency is false.

 A second option is that you are doxastically justified in believing the truth about what rationality requires. This option is compatible with Sufficiency, but implausible. Even assuming you have propositional justification for the truth, you also have apparent defeaters for the true propositions, meaning that you lack a sound deliberative route from that justification to justified beliefs based on that justification.

A third option, also compatible with Sufficiency and also implausible, is that you are doxastically justified in suspending judgement on the matter of what rationality requires. Suspension would be appropriate if it was clear to you that you had roughly equal amounts of evidence on either side—but it is not. You have what seems like strong evidence for a false belief about what is required, and propositional justification that you are unable to make use of. This does not add up to roughly equal evidence on either side.

A final option, perhaps, is that you cannot be doxastically justified in believing anything in the neighbourhood of what rationality requires. However, this is just a restatement of the Impossibility Thesis. So, the only plausible option seems to be the first one, but if this is right then Sufficiency is false.

Even worse, regardless of which option is correct, the propositional justification that Assets says we have plays very little role in determining what you should believe, given your situation. This suggests that Sufficiency is false—Assets cannot rule out evidential situations that require false beliefs in what rationality requires - they are inert. We can illustrate the inertia of Assets by comparing agents in good and bad epistemic situations, but who, according to Assets, have the same propositional justification. Consider a student, Anita, who takes Logic 101, and receives the same misleading evidence as you. Anita studies well, follows the arguments where they lead, and, like you, acquires the false belief that rationality requires contradictory belief. Compare another student, Bertha, who takes a different class. Bertha is luckier— her class teaches the true theory of what rationality requires. She also studies well and follows the arguments where they lead. She acquires true beliefs about what rationality requires.

Assets says that both Anita and Bertha have the same propositional justification about what rationality requires. If this is true, it does not seem to make much of a difference to their epistemic situations. Their propositional justification, in so far as they have it, is epistemically inert. 

Anita’s alleged propositional justification for the truth about what rationality requires is of no help to her deliberation—she lacks a viable deliberative route to doxastically justified belief in this true view. To reason her way out of her situation and towards the truth, Anita would need to rationally dismiss the misleading evidence for the false view. It is not reasonable to expect her to do this. Anita’s indefeasible propositional justification plays no role in determining what she should believe, given her evidence. It is inert. 

Nor is Bertha’s propositional justification of any help to her. Bertha studies the true theory of what rationality requires. Assets says that Bertha also has indefeasible propositional justification for the truth about what rationality requires. She also believes the truth about what rationality requires. However, it is unlikely that her belief is based on the evidence that Assets postulates. This evidence, we are told, supports the truth about what rationality requires to a maximal degree, in a way that is immune from the arguments of dialetheists. It is not clear that this would be comprehensible by a beginner. So, even though Bertha believes what is supported by her propositional justification, it is not clear that she does so on the basis of it. So, Assets would seem to be inert even in the good cases—even here they are irrelevant to what the agent ought to believe given her evidential situation.^39^ Without something that connects us epistemically to our allegedly vast store of propositional justification, the evidence that Assets postulates can play no epistemic role in either good or bad cases—it is inert. This makes it insufficient to rule out the possibility of evidential situations that support false belief about what rationality requires.

## Theoretical Upshots

Rejecting the Impossibility Thesis incurs the burden of tackling head on the problem of inconsistent requirements . In this section, I briefly mention three avenues for resolving the inconsistency without resorting to the Impossibility Thesis.

One option is to simply accept the inconsistency as an epistemic dilemma.^40^ Another is to make distinctions. For example, we might think that the apparent inconsistency arises from a failure to disambiguate two distinct sources of requirements, perhaps requirements of subjective and objective rationality.^41^ Disambiguating these, in Logic 101 we can understand you as objectively required to avoid believing contradictions, and also subjectively required to believe some contradictions. We might further flesh this out by arguing that ‘rationality requires’ is context-dependent - its usage requires reference to a context in order to be meaningful (Björnsson and Finlay 2010; Pittard and Worsnip 2017; Worsnip, forthcoming).^42^ This avoids the inconsistency because no single sense of rationality issues inconsistent requirements.

Alternatively, we might see the possibility of situations such as Logic 101 as support for denying that rationality requires enkratic coherence (see Lasonen-Aarnio 2014, 2020; Weatherson 2019). On this view, you would be required to follow your evidence in believing that rationality sometimes requires contradictory belief, but also required to avoid contradictory belief. This puts you in a tricky position, but at least it does not threaten the consistency of our theories of epistemic rationality. None of these options, perhaps, offers as easy an escape from the problem as that offered by the Impossibility Thesis, which simply nips it in the bud. However, they have the advantage of being compatible with the Simple View and thus avoid taking premature stances on what our evidence can support.

## Conclusion

The Simple View says that cases of apparently misleading evidence about what rationality requires are just ordinary cases of one’s evidence supporting a false belief - evidence can support false belief about any topic, including what rationality requires. The Impossibility Thesis avoids the problem of inconsistent requirements, but comes with its own problems. I argued here that we should reject the Impossibility Thesis. I argued that the routes for defending it face serious difficulties, and neither can offer as satisfactory an explanation of cases like Logic 101 as the Simple View. While his means that the problem of inconsistent requirements remains, there are reasons to think it is manageable.

## Footnotes

1 For example, Titelbaum describes them as “constraints on […] reasoning arising from consistency requirements among an agent’s attitudes, evidence, and whatever else reasoning takes into account.” (2015: 254).

2 As developed in Priest (1979, 1985, 2005)).

3 See (Hawthorne 2003; Nelkin 2000).

4 See (Christensen 2009).

5 See (Elga 2007).

6 See (Broome 1999, 2013; Brunero 2012; Kolodny 2005; Way and Whiting 2016).

7 See (Weatherson 2019; Lasonen-Aarnio 2014).

8 He characterises it as the view that epistemic rules are defeasible. See also (Kvanvig 2003, 2014; Way and Whiting 2016).

9 The argument bears some resemblance to Weatherson’s view that we ought to adjust our beliefs in light of higher-order evidence *only* when that higher-order evidence gives us information about what the evidence *actually* supports ((Weatherson 2019: 120, Ch. 8).

10 Bradley acknowledges that his position is consistent with either, though the comments he makes suggest he prefers the second (2019: 17).

11 This view would resemble Quine’s holism, on which everything is rationally revisable except for the fundamental truths of logic (Quine 1970, 1960).

12 For one thing, it makes mysterious why we should comply with rational requirements at all, since a natural answer is that rationality’s function is to guide agents towards epistemically better beliefs (see Gibbons (2013: 24), Rinard (2018: 259), Wedgwood (2017: 182)).

13 For defenders of this view, see (Alvarez 2010; Dancy 2000; Parfit 2011; Raz 1975; Skorupski 2010). Some accept this view while demanding an additional epistemic condition, for example Dancy’s claim that for a fact to provide one with a reason, it must pass through an ‘epistemic filter’. That is, it must be knowable (see Dancy 2000: 57–59).

14 Even if reasons are not facts, there will be a fact of the matter about which of the agent’s mental items count as reasons.

15 See (Austin 1961). This also applies to the legal concept of justification and excuse (see (Duff 2007)).

16 For further discussion of excusable norm violation in epistemology see (Boult 2019; Hawthorne and Srinivasan 2013; Kelp 2016; Littlejohn, forthcoming; Sutton 2007; Williamson forthcoming).

17 In fact, on some views it is neither. Some attempts to weaken Bayesianism’s commitment to logical omniscience include (Easwaran 2016; Gaifman 2004; Garber 1983; Hacking 1967; Williams 2018).

18 One approach is to emphasise a distinction between formal systems associated with rationality and good epistemic practices (Cohen (1981); Harman (1986); Russell (2017)), another, different, approach is offered by Stalnaker (1991). Some have nevertheless attempted the project of defending this unpopular logical omniscience requirement (see Smithies (2015), Wedgwood (2017)).

19 Articulating this is an unresolved issue (see Besson (2010), Russell (2017), Harman (1986)). Often, it takes the form of a search for the correct ‘bridge principles’ between truths of logic and normative epistemic principles. Macfarlane (2004) offers 36 different ones to choose from, each with their own theoretical costs and benefits.

20 Wedgwood (2017), for example, motivates a commitment to Assets in this way. Although he concedes that our access to these assets is restricted in such a way that respects human limitations, even with this restriction this implies that we have access to significantly more a priori evidence than empirical evidence.

21 Indefeasibility about a priori justification is defended by Field (2000); Ichikawa and Jarvis (2013); Kitcher (1980); Smithies (2015); Titelbaum (2015). In so far as the truths about what rationality requires are a priori, these views would also be committed to indefeasibility about justification for truths about what rationality requires.

22 For example, Wright explicitly denies this (Wright 2004a, b, 2014).

23 See (Boghossian 2003; Dummett 1974; Ichikawa and Jarvis 2013; Lewis 2004a; Peregrin 2014; Wright 2004a).

24 This is consistent with views that take us to have defeasible entitlement to believe the truth about what rationality requires (see Wright (2004a, b, 2014)).

25 This view dates back to Frege (see particularly his essay, “Logic” (1897). Ichikawa and Jarvis (2013) defend a more general version of this view for all a priori truths.

26 See Wright (2004b, 2014) on defeasible entitlements, as well as Bonjour on a priori justification (1998).

27 Defenders of Indefeasibility typically explain away apparent cases of defeat such as this by appeal to a distinction between defeat and some weaker defeat-like status. The previous section argued that we should resist this strategy.

28 This view is held by, for example, H. Field (2000); Ichikawa and Jarvis (2013); Kitcher (1980); Smithies (2015)).

29 For example, see Casullo (1988); Jeshion (2000); Summerfield (1991).

30 See (Hjortland 2017; Lewis 2004b; Maddy 2002; Priest 2016; Quine 1951; Russell 2015, 2014; Williamson 2007, 2013a, 2013b; Williamson and Armour-Garb 2017).

31 Even if anti-exceptionalist accounts of logic turn out to be incorrect in claiming that logic theories can be justified abductively, it may still be that abductive arguments are good enough evidence for non-experts.

32 It is worth noting that an appeal to ‘disabling’ is not obviously dialectically helpful in an argument from Assets to the Impossibility Thesis. Disabling offers a way to make possible rational false belief about what rationality requires. If Assets can be disabled, this offers a sense in which it could be rational to believe falsely about what rationality requires—without those Assets being defeated. However, those who defend the Impossibility Thesis typically do so as a way of denying the possibility of rational false belief about what rationality requires.

33 Assuming a Bayesian framework, according to which all logical truths are entailed by any evidence set.

34 For discussion, see Kahneman (2011); Kahneman et al. (1982); Tversky and Kahneman (1993).

35 For example, this would mean that Frege’s mistake about Basic Law V implies a limitation in his reasoning capacities.

36 See (Woods 2019a, b) for an articulation of this worry.

37 Whether it does is complicated, for discussion see (Hjortland 2017; Woods 2019a).

38 Turri identifies this as the ‘Orthodox view’ (2010: 314). Proponents of it include: Kvanvig (2003: §B1); Pollock (1986: 36-7); Swain (1979: 25)).

39 Consider also that most views that accept Assets take it to include not just what rationality requires, but also many other a priori truths of logic—both the very simple and the very complicated. However, there is a clear difference between our epistemic situation with respect to the simpler and the more complicated logical truths, but the distinction cannot be made by appeal to the propositional justification that Assets gives us. This suggests that this justification is inert—it makes no difference to what we ought to believe.

40 See (Hughes, forthcoming; Christensen 2010; Alexander 2013).

41 See Alston (1985); Feldman (1988); Gibbard (2005); Gibbons (2013); Goldman (1986); Kvanvig (1984); Pollock (1979); Schroeder (2009); Unger (1986)).

42 Alternatively, we might follow Worsnip (2018) in thinking that think that the relevant disambiguation is between evidential and coherence-based senses of `rationality requires’. See also Williamson (2017), who argues that epistemic rationality is ambiguous between a `content-orientated’ sense, according to which it is rational to believe p if and only if one’s evidence supports p; and a `disposition-orientated’ sense, according to which it is rational to believe p if and only if `in the same circumstances with the same evidence someone disposed to conform their beliefs to what their evidence supports would believe p. In other work, Williamson identifies this sense with epistemic blamelessness (forthcoming).
